# Improving reliability and accuracy of structured data extraction using a consensus large-language model approach–a use case description in multiple sclerosis

**DOI:** 10.3389/frai.2026.1658575

**Published:** 2026-02-13

**Authors:** Philip Lennart Poser, Rafael Klimas, Justus Luerweg, Emilie Reuter, Christoph Hanefeld, Ralf Gold, Anke Salmen, Jeremias Motte

**Affiliations:** 1 Department of Neurology, St. Josef-Hospital, Ruhr-University Bochum, Bochum, Germany; 2 Department of Internal Medicine, Katholisches Klinikum Bochum, Ruhr-University Bochum, Bochum, Germany

**Keywords:** data extraction, large language model, multiple sclerosis, neurology, real world evidence, structured data

## Abstract

**Background:**

The absence of standardization in the documentation of routine clinical data complicates research usage of retrospective data on a large-scale basis. Medically trained personnel is required for interpretation and conversion into a structured format making it time and cost intensive and creating a potential bias of such data. To address these challenges, we have developed a semi-automated approach for evaluating Multiple Sclerosis (MS) outpatients reports that utilizes different large-language models (LLM) and their consensus in comparison to manual evaluation.

**Methods:**

We used several commercially available LLMs by OpenAI, Anthropic and Google to create a structured output of several variables with differing complexity of 30 anonymized outpatient reports with zero-shot-learning. We added a consensus output by combining the results of three different LLMs. Over several runs, we adapted the prompt, compared the results with a reference and assessed the error rate. Any deviation from the reference was considered an error. A true-error rate was determined for the LLM consensus output and the neurology specialist output, where only content deviations are counted as errors.

**Results:**

Through 9 iterations of improving the structure and content of the prompt, we have seen a clear reduction in the error rate of the various LLMs. By creating an LLM consensus with the final prompt design, we were able to overcome a ceiling effect in reducing the error rate. With a true-error rate of 1.48%, the LLM consensus shows a similar error rate as neurologists (around 2%) in the creation of structured data.

**Discussion:**

Our method enables fast and reliable LLM-based analysis of large clinical routine data sets of varying complexity with a low technical barrier to entry. By generating an LLM consensus, we were able to considerably improve the quality of the output making it comparable to data created by neurology specialists. This approach allows large amounts of unstructured data to be analyzed in a time and cost-efficient manner. Nevertheless, the evaluation of errors in results produced by LLM remains difficult. Scientific work using such methods must continue to be subject to strict testing of the validity of the method in the future.

## 1 Introduction

The absence of structured documentation of clinical data is a relevant barrier to the collection and use of real-world data from medical care for scientific purposes. Most of the documentation of routine clinical data and findings is unstructured, e.g., medical history, daily visit documentation and diagnostic reports. In contrast, structured data are data that can be stored in a data organization tool such as a spreadsheet or a database. Both correctness of the content and correctness of the format are crucial for the scientific and statistical use of structured data. Information within routine clinical data is often not explicit, but only indirectly described. For example, real-world formulations to indicate the start of a treatment include vague time information such as “In spring 2022,” “Mid-February 2023” or “The next MRI (magnetic resonance imaging) examination is planned 6 months after start of treatment.” Assumptions between different parts of a report can be drawn with sufficient accuracy, e.g., if a person with Multiple Sclerosis (MS) has an Expanded Disability Status Scale (EDSS) score of less than 2.0, by definition there can be no restriction of walking distance, even if this is not explicitly stated elsewhere. The structure, the information provided and the style of, e.g., medicals reports depend on various factors, such as the given hospital IT infrastructure or personal habits of the physician.

Therefore, the extraction of data from clinical routine is often associated with data interpretation and conversion – so called “data transformation” and “data aggregation” – making it a complex task (Capurro et al., 2014; Adnan et al., 2020). The analysis of routine clinical data is frequently a very labor-intensive process requiring skilled human resources to review the data and convert it into a structured format (Tayefi et al., 2021). An analysis of further parameters in this context often requires a repetition of the data review augmenting the workload. The rich data source derived from clinical routine is thus under-used and usually focused on sub-cohorts of special interest for a specific research question. This may represent a relevant source of bias in the analysis of real-world data (RWD) (Sherman et al., 2016; Ehrenstein et al., 2024).

Large-language models (LLMs) such as ChatGPT (OpenAI), Claude (Anthropic), Gemini (Google), Llama (Meta) and others represent an attractive artificial intelligence (AI-) supported solution to transfer text-based data sources into structured ready-to-use data for further analysis in various applications (Dagdelen et al., 2024; Woznicki et al., 2025). Since the concept behind LLMs is the understanding and reproduction of natural language and not the output of structured data, the quality and ability to output structured data differs depending on the model and provider (Liu et al., 2024). The concept of using LLMs in the context of medical care is currently a rapidly expanding field of research (Gencer and Gencer, 2025).

We set out to establish an LLM-based approach to the analysis of real-world, unstructured outpatient reports, analyzing variables of varying complexity in the field of neurology. In this study, we tested the options for outputting structured data within different LLMs. In a second step, we tested different methods in a MS use case to enable timely assessment of large retrospective datasets in MS. We aimed for a practical, easy-to-use approach for clinician-scientists to obtain structured data and use it in a scientific or clinical context, e.g., quality assurance. Our research is therefore not intended to represent a definitive methodology for extracting structured data from medical records, but rather to help develop an appropriate approach that can be implemented in one’s own clinical research.

## 2 Methods

### 2.1 Source data

Outpatient reports in German language, generated by eight different neurologists, were extracted from the clinical information system filtering for reports from our MS clinic of visits between 01-Jan-2023 to 31-Dec-2023 of an academic hospital with neuroimmunological focus. Reports were only included if the visit and report date matched. Reports of 30 patients were randomly selected, manually anonymized and used for primary LLM prompt analysis and reiteratively used for refinement of the prompt.

### 2.2 Three-step human and LLM evaluation of source data

Data of interest were defined in a tabular form for human report evaluation. A first-generation prompt in plain language was generated to query the LLMs.

**Figure 1:**
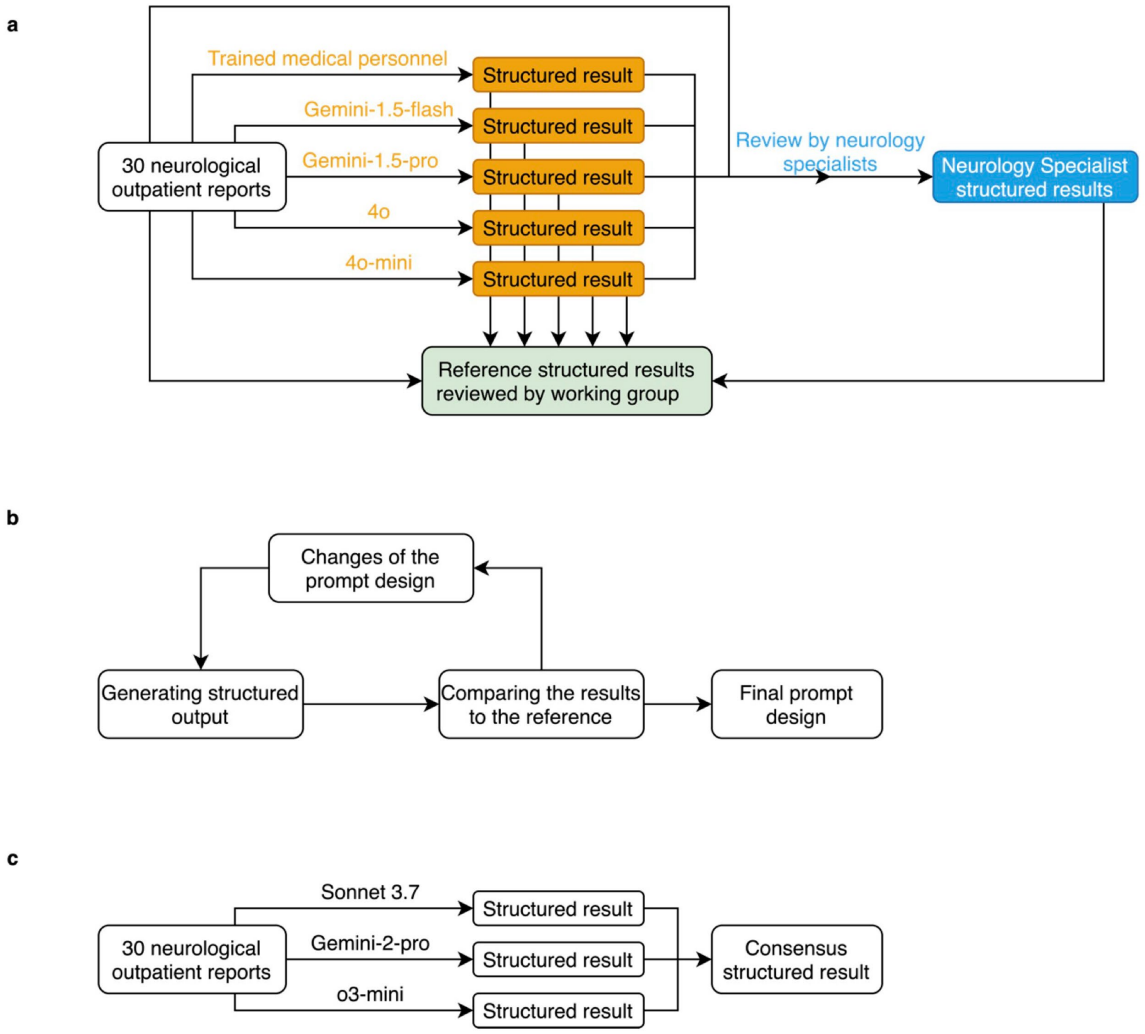
**(a)** Creation of the human reference by trained medical staff (orange) and neurology specialists (blue) and creation of the reference results (green) from the first prompt, **(b)** iterative improvement of the prompt until satisfactory result, **(c)** creation of an LLM consensus.

Medical data were analyzed in one run by trained medical personnel (TMP) and the LLMs (Figure 1a, orange path). The results of the LLMs were compared with those of the TMP by neurology specialists (NSPs) (Figure 1a, blue path). The latter extracted their structured results based on the reports, the prior human answers and LLM answers from the first prompt. These data were analyzed and a structured human reference was developed in agreement with the working group consisting of two specialists and two assistant physicians from the field of neurology (Figure 1a, green path). During the creation of the reference data, a unanimous decision was made for each variable. This reference was then used to iteratively improve the prompt (Figure 1b).

The plain language prompt was converted into a JavaScript Object Notation (JSON)-format for the structured output functions of the different LLMs, which allows responses to also be returned in JSON format.

### 2.3 Variables analyzed

We used a mix of different variable types with mixed complexity. To obtain an assessment of complexity, the working group in consensus gave a subjective assessment of the complexity of a variable, ranging from low-complex, medium-complex to high-complex. The variables analyzed were all related to the disease MS but were not specifically selected for the application of an LLM-supported evaluation. The selection of variables represents a classic evaluation of a retrospective data set in MS. The following 19 variables were examined: diagnosis (low), disease course (low), date of first manifestation (medium), date of first diagnosis (medium), current EDSS (low), oligoclonal bands (OCBs) status (low), Aquaporin-4(AQP4)-antibody (AB) status (low), myelin-oligodendocyte-glycoprotein(MOG)-AB status (low), last cranial MRI (cMRI) date (medium), cMRI activity (medium), own cMRI report interpretation (high), current immunotherapy as active substance name (low), start date of current immunotherapy (medium), previous immunotherapies as active substance name (medium), other diagnoses (medium), comedication (low), walking distance (high) and walking aid (medium).

In the evaluation of the creation of structured outputs, we also considered the following additional variables: functional scores (FS) of the EDSS, results of the Multiple Sclerosis Functional Composite (MSFC). In total, all of these latter 13 variables were only mentioned in a very small proportion of the medical records. Therefore, we excluded them from the content analysis. We also excluded the question “current neurological symptoms” from the content analysis as a reliable and distinct evaluation was not possible due to the widely differing wording of the structured results.

### 2.4 LLMs

We decided to use commercial LLMs because they allow access to models with a higher number of parameters without requiring extensive infrastructure. From our perspective, the use of commercial LLMs reflects the simplest approach to generating structured output with limited financial resources and without significant technical effort. In this study, we used the following commercially available versions of LLMs to analyze the data: claude-3-haiku-20240307 (Haiku; Anthropic), claude-3-opus-20240229 (Opus; Anthropic), claude-3-7-sonnet-20250219 (Sonnet-3.7; Anthropic), gemini-1.5-flash-002 (Gemini-1.5-flash; Google), gemini-1.5-pro-002 (Gemini-1.5-pro; Google), gemini-2.0-pro-exp-02-05 (Gemini-2-pro; Google), gpt-4o-2024-08-06 (4o; OpenAI), gpt-4o-mini-2024-07-18 (4o-mini; OpenAI) and o3-mini-2025-01-31 (o3-mini; OpenAI). The LLMs were accessed via the manufacturers’ application programming interfaces (APIs) when not stated otherwise. All requests were made with a temperature value of 0.7 and a top *p* value of 1 if applicable. Wherever possible, the data were analyzed as a batch analysis. The order of the models within this paper is based on alphabetical order and does not represent a qualitative ranking. Models are always listed in the same order as they are listed in the methods. We used the same prompt for the queries to the different LLMs. All requests for the final analysis were made between 22nd of March 2025 to 28th of March 2025.

For the consensus LLM output, we compared the output of the Sonnet-3.7, Gemini-2-pro and o3-mini models (Figure 1c). At the time of writing, these models were considered “flagship” models, offering the highest range of functionality and the best performance in the company’s own benchmarks. The idea was to use LLMs from different providers, as these have different software architectures and different training data sets. To be included, the output of two or more models had to match. If no consensus could be reached, the output was excluded from the analysis and not counted as an error.

### 2.5 Error evaluation

Errors of structure: Within the generation of a structured output, any deviation from the desired data format was considered an error. The accuracy of the content of the responses was not a factor in the evaluation of the correctness of the format.

Errors of content: Within the content evaluation, all deviations from the human consensus reference were evaluated as errors. All variables of the consensus output and the NSPs were individually compared with regard to the quality/severity of the error.

Statistical differences between the various evaluations were tested using Fisher’s exact test.

For clarity, in addition to the number of errors, we have also specified an accuracy, which is calculated as 1-(number of errors/number of variables analyzed). Based on the variables collected, it was not possible to categorize the responses of the LLMs into true positives, false positives, true negatives, and false negatives. Therefore, a conscious decision was made not to calculate precision, recall, and F1 scores, and instead to use accuracy as a measure.

### 2.6 Definition of a “true-error-rate”

The following definition was used to assess whether an error was a true-error:

- “Enumerations”: In the case of defined values from a set of possibilities, all deviations from the required answer were considered errors.
- “Dates”: In the case of dates, all data that deviated by more than 1 month from the specified date were considered incorrect. This is because wording in doctors’ letters such as “at the end of the month” leaves room for interpretation. A deviation of 1 month did not represent a deviation for the variables we analyzed that would significantly distort the outcome of a statistical analysis.
- For all other content errors, the comprehensibility of the deviation from the required answer was checked. If an answer could be verified with the help of the doctor’s letter, it was considered correct. An example of such a case is the EDSS. If the score was not clearly stated, it could be determined based on the clinical examination findings and the medical history. This leaves some room for interpretation. If the determination of an EDSS value could be verified based on the medical history and the examination findings, it was considered correct.

### 2.7 Ethical considerations

Retrospective chart analysis within our monocentric neuroimmunological registry has been approved by the ethics committee Westfalen-Lippe, Germany (registration number 2024-590-f-S). Strict data anonymization has been performed prior to any usage of the data, in particular prior to data entry into either of the LLMs.

## 3 Results

### 3.1 Generating structured outputs

A first hurdle in the use of LLMs for the evaluation of routine clinical data is the reliable generation of a structured output. There are two different ways to generate a structured output: The first way is via a plain text prompt and the second way is via a built-in function of the LLM. This can be, for example, a function calling function or a structured output function, which is supported by most of the LLM providers.

As a proof-of-concept, we first tested the generation of structured outputs using the web interface of 4o-mini. We used a text-only prompt to generate an excel spreadsheet. Our first observation was that due to the context window, we were limited in the number of medical records we could provide to the LLM. If we used a prompt at the beginning and pasted the medical records afterwards, the LLM would lose track of its task and the output would not comply with the task. We could observe that handing over the prompt with each medical record made the output much more reliable. In 2 out of 30 cases, the LLM was unable to produce an output which could be easily fixed by re-handing the task to the LLM.

As a first step, we wanted to generate a constant output of the correct columns. The columns of the spreadsheet mainly contained 3 different error types deviating from the desired format: A deviation from the column naming, a deviation from the number of columns (omitting or adding columns different from the prompt) and a deviation from the requested order. To reduce these errors, the first step was to adapt the prompt with an explicit reference to compliance with the structure. Contrary to our expectations, this increased the number of errors in 2 out of 3 error types. Overall, we were unable to generate a pure text prompt that would allow a reliable, uniformly structured output of the data (Table 1).

**Table 1:** Number of structural errors of the columns in the generation of 30 structured outputs.

Prompt	Change of column order	Omitting of columns	Incorrect naming of columns
Unmodified prompt	30	18	113
Prompt with enforced structure	21	21	147
Structured output function	0	0	0

The Prompts used were: 1. A text-only prompt without special emphasis on structured output, 2. A text-only prompt with special emphasis on structured output and 3. A prompt using the structured output function of the LLM. The test was performed with the LLM 4o-mini.

As a second step, we converted the plain text prompt to a JSON-format and used the build-in functions of different providers’ API to achieve a structured output. Many providers of LLMs offer a corresponding function to obtain a spreadsheet-like JSON output. With the help of structured output functions, we were able to drastically reduce the rate of faulty column outputs so that no more errors occurred in the structure (Table 1). From there on, we tested our prompt with the 6 most commonly used LLMs at that timepoint: 4o, 4o-mini, Gemini-1.5-pro, Gemini-1.5-flash, Opus and Haiku. All further evaluations were carried out using the structured output functions.

### 3.2 Improving the output data formats of LLMs

When generating a structured output through the methods described above we observed an important problem regarding possible further downstream analysis of the data: Data types often did not match the desired format (e.g., date formats, number formats etc.). In the next step, we therefore concentrated on generating consistent data formats.

In the first step, we started by detecting faulty data types and correcting them as best we could. The first option available for this within the structured output functions is to specify an expected data type. By setting the type of the data, we were able to reduce incorrect datatypes drastically. Although many LLMs allow to use a common schema object, not all providers support the same functions. As the LLMs “Haiku” and “Opus” do not have a specific structured output function (but can be forced to create JSON files by forcing the use of tools) and therefore were not modified by specifying a return data type, we excluded them from further analysis.

Another way to improve the creation of a structured data set is to use enumerations. With the help of these, the LLM can be given certain answer options from which to choose. In our example, we searched for diseases in the context of multiple sclerosis. By limiting the possible answers to a known set value, we were able to further reduce the number of data type errors.

As a last step, we adjusted the description of the task within the prompt and the system prompt and further emphasized the importance of sticking to the structured output.

By using these methods, we were able to reduce the rate of incorrect data formats by up to 88 percent (Table 2). Nevertheless, even after several iterations of improvement, we were not able to generate completely error-free outputs for the initially three tested models with regard to the data format.

**Table 2:** Number of errors produced in the different iterations of the improvement depending on the executor used.

Prompt modification	Haiku	Opus	Gemini-1.5-flash	Gemini-1.5-pro	Gemini-2.0-pro	4o	4o-mini	o3-mini
String-only return	100	12	81	32	–	8	9	–
Set return type	–	–	52	19	–	3	3	–
Set enum values	–	–	42	18	–	1	2	–
Revised description	–	–	37	13	–	0	1	–
Revised system prompt	–	–	34	12	0	1	1	0
Error reduction	–	–	58.02%	62.50%	–	87.50%	88.89%	–

“–“indicates that an analysis was not conducted. String-only return: Use of the structured output function without defining a specific return data format, Set return type: As before, only using a set return data format, Set enum values: As before, only using specific predefined return values, Revised description: As before, only using a different description within the prompt with regard to the data format, Revised system prompt: As before, only using a different system prompt, Error reduction: Percentage reduction in errors from the first iteration.

As a final step, we tested our JSON-format prompt with the newer LLMs 3o-mini, Gemini-2.0-pro and Sonnet-3.7. Using these models, we could not detect any errors with regards to the structure.

With the help of the first two steps, we were able to create a prompt that was able to deliver mostly consistent results in terms of output. As we could already see in the first steps that both GPT-4o-mini and Gemini-1.5-flash performed the same or worse in terms of output structure than the larger LLMs, we did not carry out the further analysis with these two. However, as they performed better in terms of structured output, we also performed our evaluations with the newer LLMs o3-mini, Gemini-2.0-experimental and Sonnet-3.7.

### 3.3 Improving and comparing output quality of LLMs

**Figure 2:**
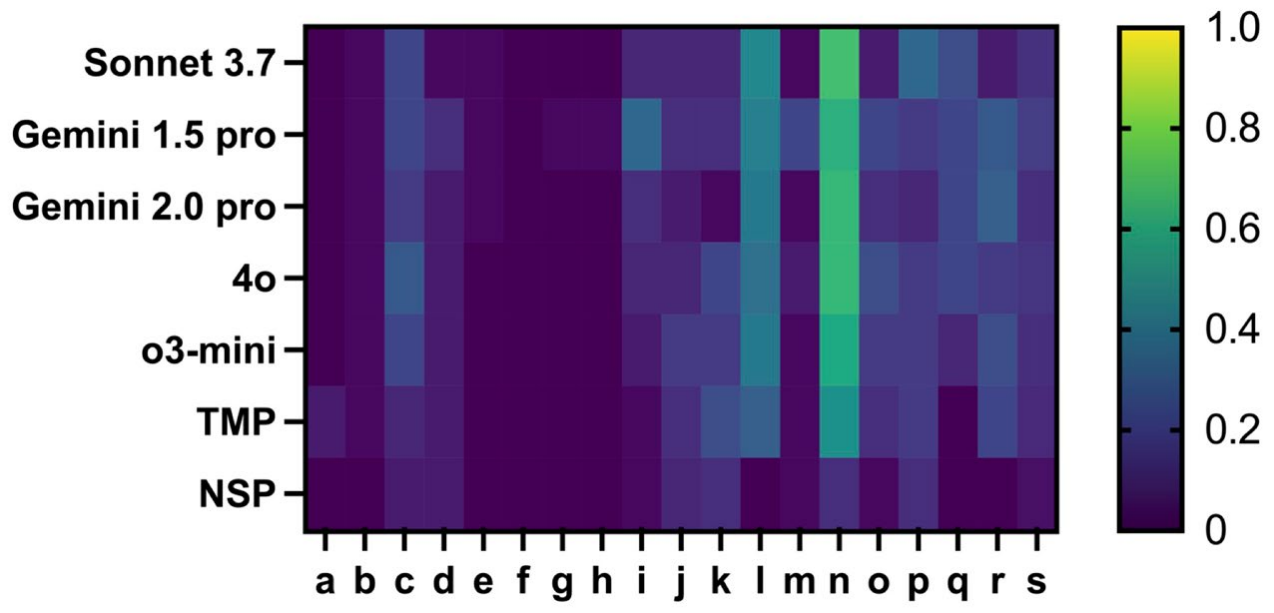
Heatmap of the error rate when evaluating the data using the first prompt in percent for the variables: a: Diagnosis, b: Disease course, c: Date of first manifestation, d: Date of first diagnosis, e: Current EDSS, f: OCB status, g: AQP4-AB status, h: MOG-AB status, i: Last cMRI date, j: cMRI activity, k: Own cMRI report interpretation, l: Current immunotherapy as active substance name, m: Start date of current immunotherapy, n: Previous immunotherapies as active substance, o: Other diagnoses, p: Comedication, q: Walking distance, r: Walking aid, s: Total errors. Especially the variables “current immunotherapy” and “previous immunotherapies” contained the most content errors in the evaluation by the LLMs.

To evaluate content quality of the outputs, we compared the different models with a human reference created for the data set. Overall, we found that errors in the TMP control and in the NSP control were lower than in the LLM evaluations. Yet, the error types within the different variables were very similar: LLMs tended to make similar errors to the human control. In particular, the variables “interpretation of cMRI activity” (incorrect interpretation), “current immunotherapy” and “previous immunotherapy” (indication of drugs instead of drug names) and “walking aid” (distinction between missing and no walking aid) caused diverging results. Interestingly, the LLM-based output highlighted certain inaccuracies in the human reference evaluation by TMPs which was revealed by the evaluation of the results by the NSPs, particularly in complex variables such as MRI interpretation and treatment timelines (Figure 2; Table 3).

**Table 3:** Number of errors depending on the prompt and LLM model used.

Variable	First prompt							Last prompt					
	Sonnet 3.7	Gemini 1.5 pro	Gemini 2.0 pro	4o	o3-mini	TMP	NSP	Sonnet 3.7	Gemini 1.5 pro	Gemini 2.0 pro	4o	o3-mini	LLM consensus
Diagnosis	0	0	0	0	2	0	0	0	0	0	0	0	0
Disease course	1	1	1	1	1	1	0	1	2	1	1	0	1
Date of first manifestation	5	6	6	6	3	8	2	5	8	7	7	3	3
Date of first diagnosis	2	1	2	4	2	2	2	2	3	2	3	2	1
Current EDSS	1	1	0	1	0	0	0	1	2	0	0	2	0
OCB status	0	0	0	0	0	0	0	0	0	0	0	0	0
AQP4-AB status	0	0	0	1	0	0	0	0	1	0	3	0	0
MOG-AB status	0	0	0	1	0	0	0	0	0	0	2	0	0
Last cMRI date	4	3	2	10	1	3	1	3	4	3	3	4	1
cMRI activity	2	3	5	4	4	3	3	3	2	2	2	1	0
Own cMRI report interpretation	1	3	5	4	7	6	4	3	2	2	5	3	1
Current immunotherapy as active substance name	12	14	12	13	9	11	0	2	2	2	2	2	2
Start date of current immunotherapy	1	1	1	6	1	2	1	1	4	2	3	2	1
Previous immunotherapies as active substance name	20	21	18	19	15	20	4	12	10	12	8	6	7
Other diagnoses	4	2	5	6	4	7	1	8	7	8	6	3	4
Comedication	3	10	5	5	5	5	4	5	6	9	7	11	4
Walking distance	6	7	3	6	0	6	0	7	7	4	9	6	5
Walking aid	9	2	7	8	6	5	0	5	4	6	6	7	6
Total errors	71	75	72	95	60	79	22	58	64	60	67	52	36
Accuracy	86.85%	86.11%	86.67%	82.41%	88.89%	85.37%	95.93%	89.26%	88.15%	88.89%	87.59%	90.37%	93.30%

Number of errors for the different variables depending on the prompt and LLM model used. In addition, accuracy is given as the proportion of correct answers out of all answers.

**Figure 3:**
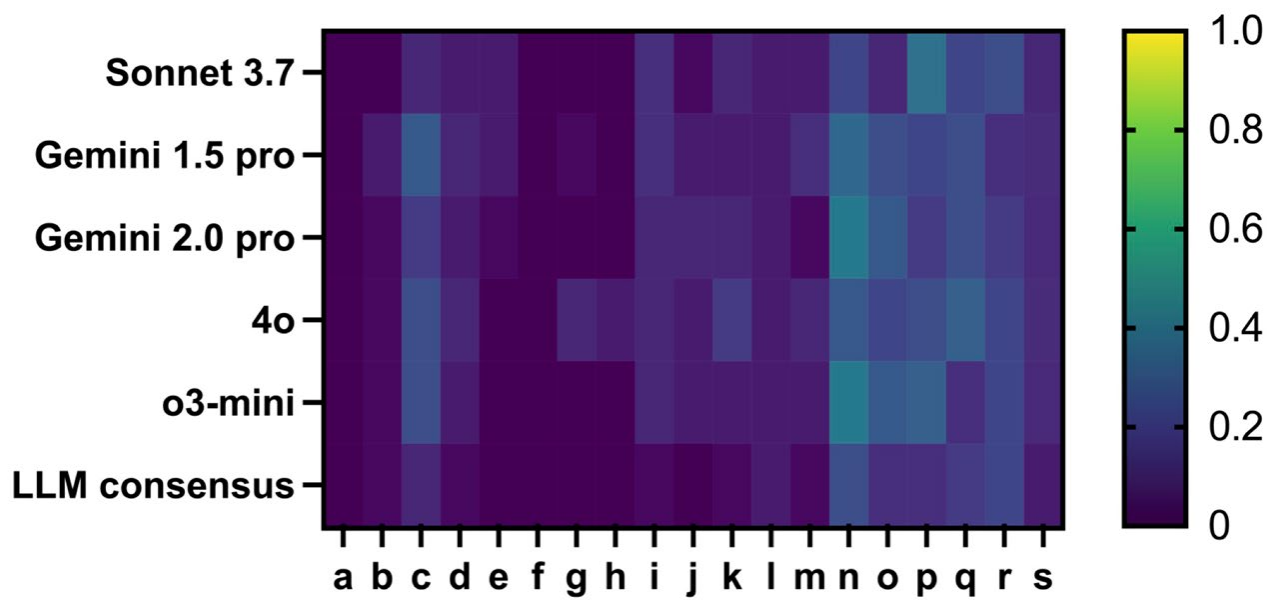
Heatmap of the error rate when evaluating the data using the second prompt in percent for the variables: a: Diagnosis, b: Disease course, c: Date of first manifestation, d: Date of first diagnosis, e: Current EDSS, f: OCB status, g: AQP4-AB status, h: MOG-AB status, i: Last cMRI date, j: cMRI activity, k: Own cMRI report interpretation, l: Current immunotherapy as active substance name, m: Start date of current immunotherapy, n: Previous immunotherapies as active substance, o: Other diagnoses, p: Comedication, q: Walking distance, r: Walking aid, s: Total errors. By adapting the prompt, a clear reduction in the content error rate was achieved, particularly in the variable “current immunotherapy” and “previous immunotherapies” can be observed in comparison to the first prompt (Figure 2). The LLM consensus generated the lowest percentage of errors in relation to the variable.

We have further improved the prompt based on this feedback from the NSPs. The main changes include: The explicit specification of criteria for cMRI interpretation, the explicit naming of active substances and their trade names with the enforced request to name only active substances and the explanation to interpret the walking distance and walking aid also in the context of the remaining examination findings and the EDSS. Thereby, we were able to reduce the number of errors by more than 25% from 15 to 11.1% in average. As this was the maximum we could achieve under several iterations of improvement, we compared an “LLM consensus” response with our reference as described in the methods part. Overall, no consensus could be found for 3 values out of a total of 540. Using this method, we were able to decrease the error rate by around another 30% compared to the best performing LLM. This method enabled us to overcome our ceiling effect and decrease the total percentage of errors to 6.7%. Nearly all variables profited from the consensus approach in terms of a reduction of the error rate (Figure 3; Table 3).

In a final step, we checked the content of all deviating answers from the LLM consensus and the NSPs individually. We were able to determine that of the 36 deviations categorized as errors, only 8 answers were actually real content-related errors. The remaining 28 answers rated as incorrect related to previously mentioned edge cases, differed only to a very small extent and could often be justified by a diverging interpretation of the unstructured data (e.g., a difference in the initial diagnosis of a few days or a few months), resulting in a true-error-rate of 1.48%. In comparison to that, out of the 22 errors by the NSPs, 11 answers were content-related errors (which were mostly caused by non-adherence to the requirements within the prompt), resulting in a true-error-rate of around 2%. The difference between the LLM consensus evaluation and the NSPs was not significant (*p* = 0.6447; odds ratio = 1.38; 95% CI 0.57–3.31).

## 4 Discussion

In our study, we were able to show that it is possible to use commercial LLMs with relatively simple means to transfer anonymized unstructured data from routine medical practice into a content-correct and reliable structured form for subsequent evaluation and clinical research. Over several iterations of improving the prompt (Figure 1b), a clear improvement in the outcome has been shown. In our small test data set, we were able to observe that LLMs are not inferior to medical professionals in the evaluation of clinical data in certain scenarios. This observation is consistent with previous studies in the area of LLM and unstructured medical data (Alkhalaf et al., 2024; Huang et al., 2024; Wiest et al., 2024; Wiest et al., 2024). These studies have so far shown that commercial LLMs are capable of evaluating routine clinical data. Previous studies tend to show higher accuracy in the evaluation of routine clinical data, although the analyses were also predominantly performed with older LLMs. One explanation for this could be that the prompt for analyzing the data was more specifically adapted to the data set. It is also conceivable that the input data was more homogeneous than our data. This would be the case, for example, with uniform diagnostic reports. However, our observation of higher accuracy of LLMs when using a consensus response is consistent with recent publications (Omar et al., 2025; MacKay et al., 2025). Our work differs from previous literature in particular in its use of a consensus approach and in the complexity and number of variables analyzed.

The first problem we encountered was the generation of a structured output. The fact that the creation of such an output is sometimes difficult and is not perceived as sufficient for many areas has already been described before (Liu et al., 2024). We have observed that the approach we used, especially with the newer LLMs, produced a reliable output. However, for automated pipelines it should be kept in mind that using the methods we used does not give 100 percent certainty of obtaining a correct return format.

In the next step, we were able to show that a significant improvement of the content of the output could be achieved by adjusting the prompt. It is important to note that in our case the prompt was adapted with consideration to the inputs. For example, edge cases were analyzed in detail and considered by formulations within the prompt. It has already been shown that the outcomes of LLMs in a clinical context depend on the level of detail of the prompt (Burford et al., 2024). At the same time a precise adjustment of the prompt also means canceling out the time advantages of the LLM (Shah, 2024). A compromise must therefore always be found between accuracy and effort. Another important aspect of these findings is that the content reliability of a prompt only applies to a specific data set: the one it was developed for. Conversely, this also means that the traceability of the data creating is less good. Another problem with the approach we have described is that adapting the prompt to the data set can cause overfitting. This can distort the results of the accuracy analysis. Adapting a prompt to the routine clinical data of a clinic also means that transferring it to other clinical data is likely to be possible only with significant adaptation, if at all. Because of these reasons we see the need for a precise and comprehensible description of the establishment of an LLM-supported evaluation of clinical data. Such an evaluation should not only be performed at the beginning but should also be done throughout the whole data generation process.

Even though our focus was not on the comparison of the different LLMs, we were able to see that especially newer LLMs tended to perform better when analyzing the unstructured data. However, we cannot say conclusively from our study how the various LLMs perform with larger data sets. There are also reports of differences of the accuracy between certain LLMs (Ntinopoulos et al., 2025). We are therefore unable to make a general statement as to which LLM should be used to evaluate medical data. Especially since we have limited ourselves to evaluating only a few selected LLMs, we cannot make any more precise statements about “the best LLM.” It would also be conceivable, for example, that different LLMs benefit from a specially tailored prompt and that using a universal prompt is not the best way to obtain the most accurate results. For reasons of feasibility, we have, for example, refrained from analyzing specialized medical LLMs such as Med-PaLM 2 or others (Singhal et al., 2025). Fine-tuning an existing model could also be a way to increase the accuracy of LLM responses for a specific dataset (Bui et al., 2025). An important finding in this context is that we were able to bridge a certain ceiling effect of correct answers through the pooled use of several LLMs from different providers introducing a consensus decision. The use of multiple LLMs for a consensus finding might hold great potential - despite the increased costs - as an approach to ensure the best possible accuracy of the data in terms of content and structure when collecting complex variables. This approach, the use of different intelligences and specializations to achieve the best outcome is ultimately borrowed from medical practice, where so-called boards (e.g., tumor board or immunoboard) are used to solve complex medical cases. To our knowledge, this approach in LLMs is not yet established. However, further studies with larger datasets are needed to confirm our hypotheses.

Another problem we identified throughout our study is the evaluation of data accuracy – particularly for variables which need interpretation. While counting outputs which deviate from a defined reference in our evaluation was a good way to improve the prompt, it also overrepresented errors defined as a deviation from a given standard, but not necessarily wrong in content. Most approaches to the use of LLMs in the healthcare context focus on diagnostics and not on the output of structured data (Meng et al., 2024; Ullah et al., 2024; Nazi and Peng, 2024). There are general guidelines for publishing with the help of LLMs (Gallifant et al., 2025). Nevertheless, we could observe that it is extremely difficult to describe the accuracy of a method sufficiently well. This difficulty in describing accuracy poses a threat to the comprehensibility of scientific data. Within the manuscript, a conscious decision was made against extensive statistical analysis. From our perspective, *p*-values can be misleading in the context of LLM evaluations. A specific adaptation of a prompt to a data set will probably always lead to high accuracy for that specific data set and result in a positive outcome in a statistical evaluation. However, this does not automatically mean that the results can be transferred to other data. Additionally, the use of LLMs bears several limitations and may introduce a qualitative bias into scientific data analyses due to a lack of accountability and transparency (Clusmann et al., 2023). Repetitive inquiries may result in diverging output data due to performance fluctuation or updates. Hallucinations pose a risk of generating incorrect data (Qiu et al., 2024). The state of knowledge of LLMs is limited to its training data and usually does not contain all latest scientific findings and LLMs are usually operated by commercial providers associated with risks of data protection and improper further data usage.

One issue that arises from our evaluation is the limited proven transferability. Using a small data set from a specific cohort, we were able to show that a consensus-based evaluation of physician letters is not inferior to a manual evaluation. The data and variables were not specifically selected for evaluation using LLM. Although it is conceivable in principle that a consensus LLM-based evaluation could also deliver better results in the context of other diseases and data sets, we cannot substantiate this thesis with our current results.

By using LLMs, we were able to reduce the time and costs required. An analysis of 30 medical records using batch analysis cost less than one dollar. At the same time, the cumulative time required for analysis by medical staff for all medical records was reduced from around 5 h to just a few minutes. Nevertheless, it should be borne in mind that the use of a consensus model multiplies the costs. The use of three or more LLMs also means triple or higher costs. Despite the increased costs, the use of consensus in our application case was a significantly cheaper method than manual evaluation. Overall LLMs show great potential for the evaluation of unstructured medical data but should be used with caution and under a critical view, especially when applied in complex situations.

## 5 Limitations

Although we were able to test a range of different LLMs, this is only a small sample of the options currently available. Since our test data set was limited to 30 records, we cannot make a definitive statement about the best possible use of LLMs for the evaluation of unstructured medical data. A significantly higher number of records would be necessary to make a definitive statement about the comparison of different LLMs. Likewise, a significant increase in the number of different LLMs analyzed would be necessary. The data comes from a single-center analysis, which makes it difficult to compare with other clinics and diseases. Likewise, we only included MS-specific variables in our study. In addition, the iterative prompt improvement approach may result in overfitting of our prompt to the dataset. Our selection of variables represents only a minimal excerpt of the potentially collectible data. Therefore studies with larger data sets are necessary to confirm our hypotheses and observations.

## Data Availability

The raw data supporting the conclusions of this article will be made available by the authors, without undue reservation.
