# Supplementary material for: Improving reliability and accuracy of structured data extraction using a consensus large-language model approach–a use case description in multiple sclerosis
Source: Front Artif Intell. 2026 Feb 13;9:1658575. doi: 10.3389/frai.2026.1658575 (PMC12946029; doi:10.3389/frai.2026.1658575)
Supplement: Supplementary file 1 [file Data_Sheet_1.pdf]

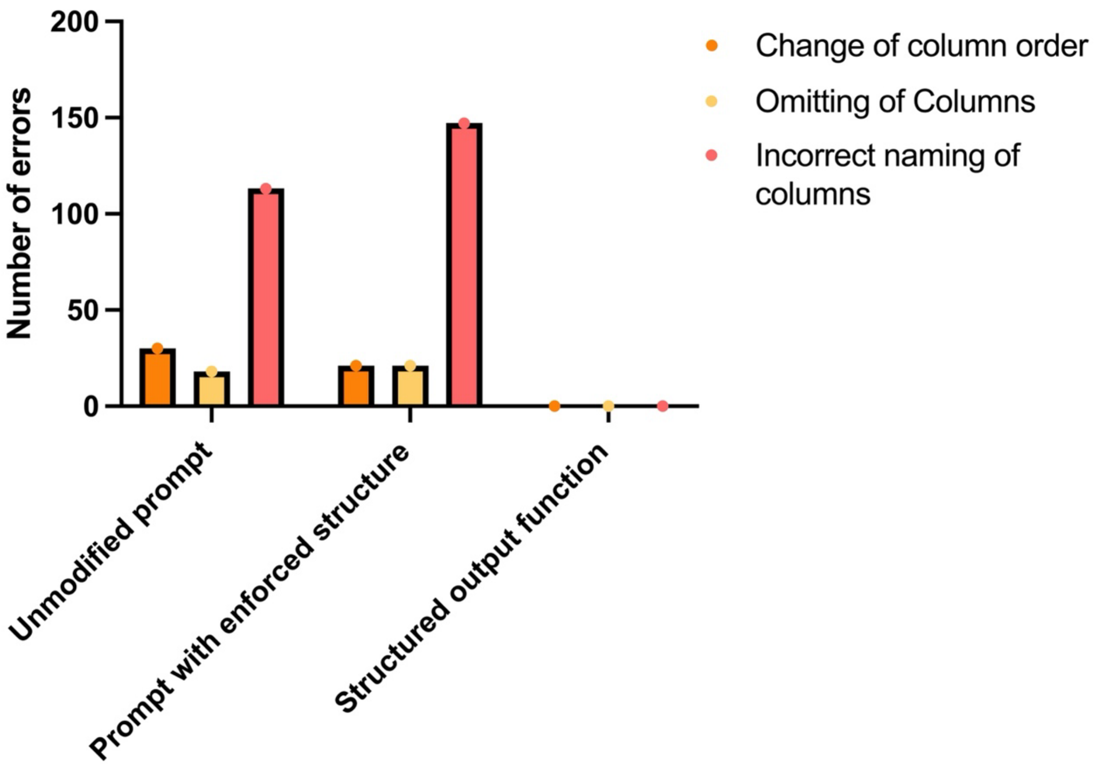

Supplemental Figure 1: Number of structural errors in the creation of 30 structured outputs using different methods for creating structured outputs (compare Table 1)
