# Supplementary material for: Improving reliability and accuracy of structured data extraction using a consensus large-language model approach–a use case description in multiple sclerosis
Source: Front Artif Intell. 2026 Feb 13;9:1658575. doi: 10.3389/frai.2026.1658575 (PMC12946029; doi:10.3389/frai.2026.1658575)
Supplement: Supplementary file 2 [file Data_Sheet_2.pdf]

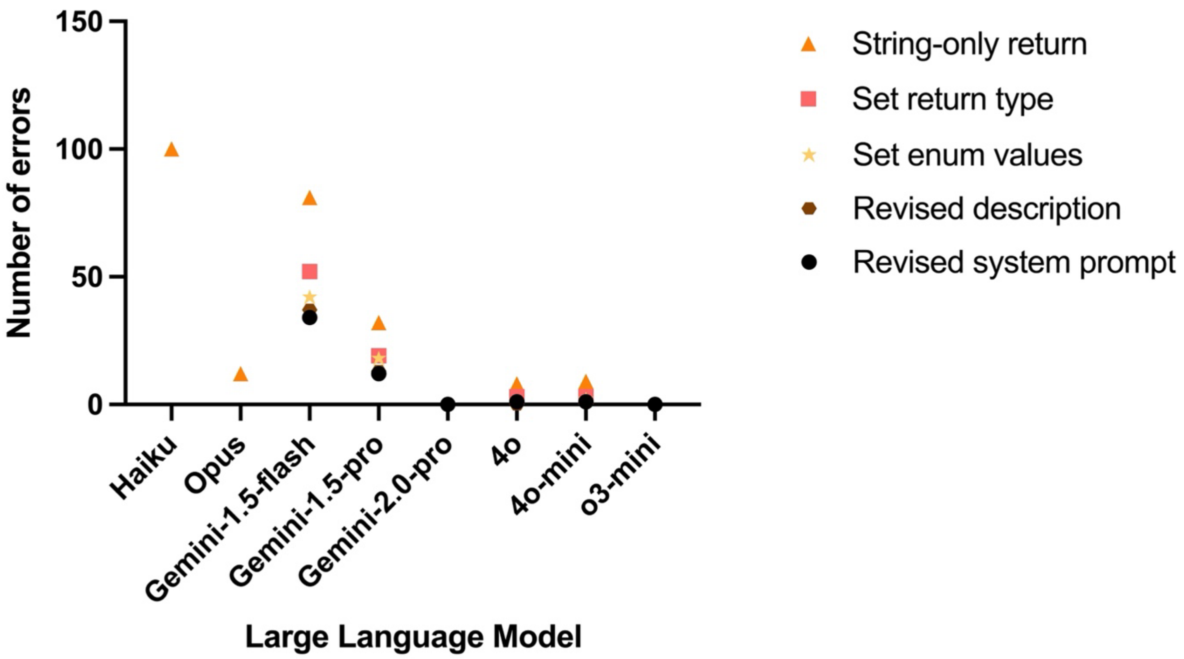

Supplemental Figure 2: Number of errors when using different prompt adjustments to generate structured outputs and when using different large language models (see Table 2)
