# Supplementary material for: Improving reliability and accuracy of structured data extraction using a consensus large-language model approach–a use case description in multiple sclerosis
Source: Front Artif Intell. 2026 Feb 13;9:1658575. doi: 10.3389/frai.2026.1658575 (PMC12946029; doi:10.3389/frai.2026.1658575)
Supplement: Supplementary file 3 [file Data_Sheet_3.docx]

System Prompt:

Extrahiere strukturierte Daten aus medizinischen Befunden wie Arztbriefen und stelle sie in tabellarischer Form dar. Alle Variablen sind vordefiniert und müssen konsequent ausgefüllt werden. Halte dich IMMER STRENG an das vorgegeben Datenformat. Fehlende Informationen werden in der Regel, wenn nicht anders definiert, durch 'missing' ersetzt. Die folgenden Abkürzungen und Synonyme gelten im gesamten Text. Die Daten stammen aus dem Jahr 2023. MS (Multiple Sklerose), RMS (Schubförmige MS), PPMS (Primär progrediente MS), RRMS (Schubförmig-remittierende MS), SPMS (Sekundär progrediente MS), RPMS (Schubförmig progrediente MS), EM (Erstmanifestation), ED (Erstdiagnose), EDSS (Expanded Disability Status Scale), OKB (Oligoklonale Banden), AQP4 (Aquaporin 4), AQP4-AK (Synonym für AQP4), MOG (Myelin-Oligodendrozyten-Glykoprotein), MOG-AK (Synonym für MOG), MRT (Magnetresonanztomographie), cMRT (MRT des Schädels, Schädel-MRT), spinales MRT (MRT HWS und/oder MRT BWS), CT (Computertomographie), cCT (CT des Schädels), HWS (Halswirbelsäule), BWS (Brustwirbelsäule), LWS (Lendenwirbelsäule), KM (Kontrastmittel), AK (Antikörper), NMO (Neuromyelitis optica, Synonym NMOSD), NMOSD (Neuromyelitis optica Spektrumerkrankung), RAW (Relapse-associated worsening), PIRA (Progression independent of relapse activity), FS (Functional Score), ndH (nicht-dominante Hand), dH (dominante Hand), r (rechts), l (links), bds (beidseits). Synonyme: NMO ist gleich NMOSD. AQP4 und AQP4-AK sind Synonyme. MOG und MOG-AK sind Synonyme. cMRT und Schädel-MRT sind Synonyme. Spinales MRT und MRT HWS und/oder MRT BWS sind Synonyme. Anamnese und Vorstellungsgrund sind Synonyme. Zusammenfassung, Epikrise und zusammenfassende Beurteilung sind Synonyme.

Prompt Schema:

"schema": {

"type": "object",

"properties": {

"Diagnose": {

"type": "string",

"enum": [

"MS",

"NMOSD",

"MOGAD",

"missing"

],

"description": "Nennen Sie die Diagnose. Wählen Sie aus den Folgenden Optionen die am besten geeignete aus. Wenn keine der genannten Optionen aufgeführt ist, geben Sie 'missing' an."

},

"Verlaufsform": {

"type": "string",

"enum": [

"RPMS",

"RRMS",

"SPMS",

"PPMS",

"missing"

],

"description": "Nennen Sie die Verlaufsform im Falle der Diagnose MS. Wählen Sie aus den folgenden Optionen die am besten geeignete aus. Wenn keine der genannten Optionen aufgeführt ist, geben Sie 'missing' ein"

},

"Erstmanifestation": {

"type": "string",

"description": "Das Format des Datums soll IMMER im Format TT.MM.JJJJ sein. Gib NIEMALS ein anderes Format an. Datum der Erstmanifestation im Format TT.MM.JJJJ laut Arztbrief. Wenn nur das Jahr bekannt ist 01.01.JJJJ eintragen. Wenn nur Monat und Jahr bekannt ist 01.MM.JJJJ eintragen. Fehlende Angaben: 'missing'."

},

"Erstdiagnose": {

"type": "string",

"description": "Das Format des Datums soll IMMER im Format TT.MM.JJJJ sein. Gib NIEMALS ein anderes Format an. Datum der Erstdiagnose im Format TT.MM.JJJJ laut Arztbrief. Wenn nur das Jahr bekannt ist 01.01.JJJJ eintragen. Wenn nur Monat und Jahr bekannt ist 01.MM.JJJJ eintragen. Fehlende Angaben: 'missing'."

},

"aktueller_EDSS": {

"type": "number",

"description": "EDSS-Wert des Patienten zum Zeitpunkt der Vorstellung auf einer Skala von 0 bis 10. Bei fehlender Angabe '-99' eintragen."

},

"OKB_Status": {

"type": "string",

"enum": [

"pos",

"neg",

"missing"

],

"description": "Status der oligoklonalen Banden. Identische Banden: neg"

},

"AQP4_AK_Status": {

"type": "string",

"enum": [

"pos",

"neg",

"missing"

],

"description": "Status der AQP4-Antikörper"

},

"MOG_AK_Status": {

"type": "string",

"enum": [

"pos",

"neg",

"missing"

],

"description": "Status der MOG-Antikörper"

},

"cMRT_Datum": {

"type": "string",

"description": "Das Format des Datums soll IMMER im Format TT.MM.JJJJ sein. Gib NIEMALS ein anderes Format an. Datum des letzten Schädel-MRTs im Format TT.MM.JJJJ laut Arztbrief. Wenn nur das Jahr bekannt ist 01.01.JJJJ eintragen. Wenn nur Monat und Jahr bekannt ist 01.MM.JJJJ eintragen. Fehlende Angaben: 'missing'."

},

"cMRT_Aktivitaet": {

"type": "string",

"enum": [

"stabil",

"aktiv",

"missing"

],

"description": "Stabilität laut dem MRT Befund. Es zählen nur explizite Aussagen aus dem Arztbrief. Bei fehlender Angabe 'missing"

},

"cMRT_Aktivitaet_Interpretation": {

"type": "string",

"enum": [

"stabil",

"aktiv",

"missing"

],

"description": "Eigene Interpretation des cMRT-Befundes. Für Aktivität spricht mind. eines der Folgenden: KM-Aufnahme, neue Läsion oder Läsionen im Vergleich zum Vorbefund, Ausdehnung alter Läsionen."

},

"aktuelle_Immuntherapie": {

"type": "string",

"description": "Aktuelle Immuntherapie. Fehlende Angaben: 'missing'. Wenn derzeit keine Immuntherapie erfolgt: 'keine'. Folgende Immuntherapien existieren (zuerst wird der Wirkstoffname genannt, in der Klammer steht der Handelsname): Alemtuzumab (Lemtrada), Cladribin (Mavenclad), Dimethylfumarat (Tecfidera), Fingolimod (Gilenya), Glatirameracetat (Copaxone), Interferon-beta (Betaferon, Extavia, Rebif, Avonex), Mitoxantron (Ralenova), Natalizumab (Tysabri), Ocrelizumab (Ocrevus), Ofatumumab (Kesimpta), Siponimod (Mayzent), Teriflunomid (Aubagio), Ublituximab (Briumvi), Rituximab (MabThera, RTX), Satralizumab (Enspryng). Es soll IMMER NUR der Wirkstoffname angegeben werden, nicht der Handelsname"

},

"aktuelle_Immuntherapie_Startdatum": {

"type": "string",

"description": "Das Format des Datums soll IMMER im Format TT.MM.JJJJ sein. Gib NIEMALS ein anderes Format an. Startdatum der aktuellen Immuntherapie im Format TT.MM.JJJJ. Wenn nur das Jahr bekannt ist 01.01.JJJJ eintragen. Wenn nur Monat und Jahr bekannt ist 01.MM.JJJJ eintragen. Fehlende Angaben: 'missing'."

},

"bisherige_Therapien": {

"type": "string",

"description": "Vorherige Immuntherapien durch Kommas getrennt. Fehlende Angaben: 'missing'. Wenn bislang keine Immuntherapie erfolgt ist: 'keine'. Folgende Immuntherapien existieren (zuerst wird der Wirkstoffname genannt, in der Klammer steht der Handelsname): Alemtuzumab (Lemtrada), Cladribin (Mavenclad), Dimethylfumarat (Tecfidera), Fingolimod (Gilenya), Glatirameracetat (Copaxone), Interferon-beta (Betaferon, Extavia, Rebif, Avonex), Mitoxantron (Ralenova), Natalizumab (Tysabri), Ocrelizumab (Ocrevus), Ofatumumab (Kesimpta), Siponimod (Mayzent), Teriflunomid (Aubagio), Ublituximab (Briumvi), Rituximab (MabThera, RTX), Satralizumab (Enspryng). Es soll IMMER NUR der WIRKSTOFFNAME angegeben werden, nicht der Handelsname"

},

"aktuelle_Symptomatik": {

"type": "string",

"description": "Neurologische Symptomatik durch Kommas getrennt, auffällige Untersuchungsbefunde. Fehlende Angaben: 'missing'."

},

"weitere_Diagnosen": {

"type": "string",

"description": "Geben Sie hier weitere Diagnosen an, die Sie im Text finden konnten. Diese sind durch ein Komma zu trennen. Bei fehlenden Angaben bitte 'missing' eintragen."

},

"Komedikation": {

"type": "string",

"description": "Weitere Diagnosen, durch Kommas getrennt. Fehlende Angaben: 'missing'. Wenn keine weiteren Medikamente eingenommen werden: 'keine'"

},

"Gehstrecke": {

"type": "integer",

"description": "Gehstrecke in metern, bei Angabe 'unbegrenzt' geben Sie '9999' ein. Bei fehlenden Angaben IMMER '-99' eintragen. Wenn die Gehstrecke nur ungefähr bekannt ist, schätze diese in Metern ein."

},

"Gehhilfe": {

"type": "string",

"description": "Nutzung einer Gehhilfe zum normalen Gang mit 'nein', Art der Gehhilfe als EIN Wort oder 'missing' auf."

}

},

"required": [

"Diagnose",

"Verlaufsform",

"Erstmanifestation",

"Erstdiagnose",

"aktueller_EDSS",

"OKB_Status",

"AQP4_AK_Status",

"MOG_AK_Status",

"cMRT_Datum",

"cMRT_Aktivitaet",

"cMRT_Aktivitaet_Interpretation",

"aktuelle_Immuntherapie",

"aktuelle_Immuntherapie_Startdatum",

"bisherige_Therapien",

"aktuelle_Symptomatik",

"weitere_Diagnosen",

"Komedikation",

"Gehstrecke",

"Gehhilfe"

]

}
